# Parity influences the severity of ACPA-negative early rheumatoid arthritis: a cohort study based on the Swedish EIRA material

**DOI:** 10.1186/s13075-015-0869-x

**Published:** 2015-12-12

**Authors:** Mitra Pikwer, Cecilia Orellana, Henrik Källberg, Andreas Pikwer, Carl Turesson, Lars Klareskog, Lars Alfredsson, Saedis Saevarsdottir, Camilla Bengtsson

**Affiliations:** Rheumatology Unit, Mälarsjukhuset Hospital, Eskilstuna, Sweden; Institute of Environmental Medicine, Karolinska Institutet, Stockholm, Sweden; Centre for Clinical Research Sörmland, Uppsala University, Eskilstuna, Sweden; Rheumatology, Department of Clinical Sciences, Malmö, Lund University, Malmö, Sweden; Rheumatology Unit, Department of Medicine, Karolinska Institutet and Karolinska University Hospital Solna, Stockholm, Sweden; Centre of Occupational and Environmental Medicine, Stockholm County Council, Stockholm, Sweden

**Keywords:** Rheumatoid arthritis, Parity, Clinical outcome, Epidemiology, Hormonal factors

## Abstract

**Background:**

In women with rheumatoid arthritis (RA) it has been observed that during pregnancy a majority of patients experience amelioration, but after delivery a relapse of the disease is common. However, there are few studies, with diverging results, addressing the effect of parity on the severity of RA over time. Our aim was to explore the impact of parity, with stratification for anti-citrullinated protein antibody (ACPA) status as well as for onset during reproductive age or not.

**Methods:**

Female RA cases aged 18–70 years were recruited for the Epidemiological Investigation of Rheumatoid Arthritis (EIRA). Information on disease severity (the health assessment questionnaire (HAQ) and the disease activity score 28 (DAS28)) was retrieved from the Swedish Rheumatology Quality Register at inclusion and 3, 6, 12 and 24 months after diagnosis. Mixed models were used to compare mean DAS28 and HAQ scores over time in parous and nulliparous women. Mean differences at individual follow-up visits were compared using analysis of covariance. The odds of having DAS28 or HAQ above the median in parous verus nulliparous women were estimated in logistic regression models.

**Results:**

A total of 1237 female cases (mean age 51 years, 65 % ACPA-positive) were included. ACPA-negative parous women, aged 18–44 years, had on average 1.17 units higher DAS28 (*p* < 0.001) and 0.43 units higher HAQ score (*p* < 0.001) compared to nulliparous women during the follow-up time, adjusted for age. In this subgroup, the average DAS28 and HAQ scores were significantly higher in parous women at all follow-up time points. Younger parous ACPA-negative women were significantly more likely to have DAS28 and HAQ values above the median compared to nulliparous women at all follow-up visits. No association between parity and severity of ACPA-positive disease was observed.

**Conclusions:**

Parity was a predictor of a more severe RA among ACPA-negative younger women, which might indicate that immunomodulatory changes during and after pregnancy affect RA severity, in particular for the ACPA-negative RA phenotype.

## Background

Female sex and older age are known risk factors for rheumatoid arthritis (RA). The disease is, however, heterogeneous, and a common division occurs between the presence/absence of autoantibodies to citrullinated peptide antigens (ACPA) where ACPA-positive disease generally has a worse outcome. Established genetic (e.g., HLA-DRB1 SE alleles) and environmental (e.g., smoking) risk factors are also predominantly associated with the risk of ACPA-positive RA [1, 2].

Previous findings on the impact of parity on RA development have shown a reduction in RA incidence during pregnancy [3] and an increased risk post-partum [4]. In contrast, parity in the long run seems to have no association [5–7], or even reduced risk of RA [8]. In a recent study we reported that parous women of reproductive age had an increased risk of ACPA-negative RA, and that this increased risk was seen mainly in women who gave birth during the year of symptom onset. There was no association with risk of ACPA-positive RA [9].

In women with RA, it has been observed that during pregnancy a majority of patients experience amelioration [10, 11]. After delivery a relapse of RA is common, especially in women who breastfeed [12, 13]. There are only a few studies with diverging results regarding the effect of parity on the severity of RA (or inflammatory polyarthritis) over time [12–15]. Here, in an unselected population-based cohort of early RA with extensive information about lifestyle and environmental factors, in a country with even access to healthcare, our aim was to explore the impact of parity on the severity of RA, with stratification for ACPA status as well as between those in reproductive age and those who were older at disease onset.

## Methods

### The EIRA study

We studied female incident RA cases aged 18–70 years, included between 1996 and 2009 in the Swedish Epidemiological Investigation of Rheumatoid Arthritis (EIRA) study, a population-based case–control study performed in parts of Sweden. EIRA has been described more extensively elsewhere [16]. All patients included were diagnosed by a rheumatologist and fulfilled the American College of Rheumatology 1987 criteria for RA [17]. The mean duration from symptom onset was 7 months. All participants gave informed consent and the study was approved by the Ethical Review Board at the Karolinska Institute.

### Data collection

An extensive questionnaire was used to collect information on lifestyle and environmental factors, including parity. Of 2162 identified cases, 2063 (95 %) answered the questionnaire. In addition, the participating cases provided blood samples for serological and genetic analyses.

Women were classified as parous (those who had given birth before or during the year of diagnosis), or nulliparous at diagnosis. Information about parity history after diagnosis was not available. In total, 44 cases lacked information on parity.

### Antibody assays

Immunoscan-RA Mark2 ELISA test (Euro-Diagnostica, Malmo, Sweden) was used to determine ACPA status [18]. The cut-off was set to 25 U/ml for ACPA-positive RA. Information about ACPA status was missing in 26 cases.

### Clinical outcome

Clinical data were captured from the Swedish Rheumatology Quality Register (SRQ) until 2010. The register includes information about disease activity and disability at predefined time points. The process of capturing data on EIRA patients from SRQ was described previously [19]. There were 746 patients in EIRA who were not included in SRQ at inclusion in EIRA (baseline) or had missing disease activity parameters at baseline. Some of the patients had not yet reached the follow-up time points for data collection in SRQ (see below).

We focused on two outcome measures: the disease activity score 28 (DAS28) and the health assessment questionnaire (HAQ). These were evaluated at diagnosis and at the 3-, 6-, 12- and 24-month follow-up visits in SRQ. Not all repeated data were complete for DAS28 versus HAQ (0 versus 3 % missing at inclusion, 19 versus 22 % missing at 3 months, 34 versus 36 % missing at 6 months, 10 versus 12 % missing at 12 months and 27 versus 29 % missing at 24 months).

### Statistics

We stratified the cases into ACPA-negative/ACPA-positive disease, and age groups (18–44 and 45–70 years) [9]. We analyzed the data in three ways: 1) To compare mean DAS28 and HAQ scores over time in parous and nulliparous women we used mixed models with a first-order heterogeneous autoregressive correlation structure to allow for time-dependent variance and declining correlation between time points further away from each other. 2) Analysis of covariance was used to analyze differences in mean DAS28 and HAQ scores at the different time points between parous and nulliparous women, adjusted for age at inclusion in EIRA. We further adjusted individually for potential confounders (ever/never smoking; university/no university degree; ever/never use of oral contraceptives; and area of living). Questions about breastfeeding were added to the questionnaire in 2006 and a subanalysis on patients included in years 2006–2009 was performed with additional adjustment for ever/never breastfeeding amongst parous women. Adjustments for the abovementioned factors only marginally altered the results and were therefore not retained in the analyses. 3) Logistic regression was performed to obtain odds ratios with 95 % confidence interval (CI) of having DAS28 or HAQ above the median at the different time points for parous women compared to nulliparous women.

All analysis was performed using Statistical Package for the Social Sciences22 IBM Corporation 1 New Orchard Road Armonk, New York 10504-1722 United States http://www-01.ibm.com/software/analytics/spss/products/statistics/.

## Results

In total, 1237 female cases with concurrent information on parity and ACPA status in EIRA were also included in the SRQ. Mean age at inclusion in EIRA was 52 years. The mean time between first symptoms and RA diagnosis was approximately 6 months, and no differences were found according to ACPA status. In all, 82 % had ever given birth to a child before diagnosis and 65 % were ACPA positive (Table 1).

**Table 1:** Baseline characteristics of incident rheumatoid arthrits cases included in EIRA

	All women (n = 1237)	ACPA negative		ACPA positive	
		Aged 18–44 (n = 103)	Aged 45–70 (n = 331)	Aged 18–44 (n = 230)	Aged 45–70 (n = 573)
Mean age, years (SD)	51.5 (12.8)	32.9 (7.0)	58.8 (6.6)	34.3 (7.4)	57.6 (7.0)
Parous, n (%)	1009 (81.6)	68 (66.0)	292 (88.2)	135 (58.7)	514 (89.7)
Nulliparous, n (%)	228 (18.4)	35 (34.0)	39 (11.8)	95 (41.3)	59 (10.3)
Mean time between last birth to symptom onset, years (SD)	24.7 (12.1)	6.8 (4.7)	29.8 (8.1)	7.2 (5.4)	28.8 (9.2)
Mean age at first birth, years (SD)	24 (4.9)	26 (5.3)	24 (4.9)	26 (4.6)	24 (4.8)
Mean number of children (SD)	2.2 (0.9)	1.9 (0.5)	2.4 (0.9)	2.3 (1.2)	2.2 (1.0)
Median DAS28 at inclusion, years (SD)	5.4 (1.2)	5.2 (1.1)	5.6 (1.2)	5.3 (1.1)	5.4 (1.2)
Median HAQ at inclusion (SD)	1.0 (0.6)	1.0 (0.5)	1.1 (0.6)	1.0 (0.6)	1.1 (0.6)
Ever smokers, n (%)	823 (66.7)	59 (57.3)	202 (61.2)	126 (55.0)	436 (76.2)
University degree, n (%)	318 (25.7)	31 (30.1)	85 (25.7)	82 (35.7)	120 (21.0)
Ever use of oral contraceptives before symptom onset, n (%)	807 (65.6)	89 (89.0)	195 (59.1)	185 (81.1)	338 (59.1)
Received DMARD at inclusion, n (%)	1109 (87)	91 (87)	304 (90)	190 (87)	522 (89)
Received prednisolone at inclusion, n (%)	476 (37)	32 (29)	133 (38)	70 (31)	241 (40)

*ACPA* Anti-citrullinated protein antibody, *DAS28* Disease activity score 28, *DMARD* Disease-modifying antirheumatic drug, *EIRA* Epidemiological Investigation of Rheumatoid Arthritis, *HAQ* Health assessment questionnaire, *SD* Standard deviation

### Disease severity over time: mixed models analysis

Over time, parous women aged 18–44 years had on average higher DAS28 (mean difference 1.17, 95 % CI 0.65 to 1.68) and higher HAQ (mean difference 0.43, 95 % CI 0.20 to 0.66) scores compared to nulliparous women at each follow-up (Table 2). Among women who developed ACPA-negative disease at older age, parous women tended to have a lower DAS28 (Table 2).

**Table 2:** Adjusted mean differences in clinical outcomes over the first 2 years between nulliparous and parous women

	DAS28	HAQ
	Beta (95 % CI), *p*-value	Beta (95 % CI), *p*-value
All patients	0.11 (−0.05 to 0.27), *p* = 0.17	0.06 (−0.00 to 0.13), *p* = 0.07
ACPA negative		
Aged 18–44	1.17 (0.65 to 1.68), *p* < 0.001	0.43 (0.20 to 0.66), *p* < 0.001
Aged 45–70	−0.26 (−0.62 to 0.09), *p* = 0.14	−0.06 (−0.20 to 0.09), *p* = 0.46
ACPA positive		
Aged 18–44	0.06 (−0.25 to 0.38), *p* = 0.69	0.07 (−0.05 to 0.19), *p* = 0.27
Aged 45–70	0.08 (−0.20 to 0.36), *p* = 0.57	0.02 (−0.10 to 0.14), *p* = 0.70

Mixed models adjusted for age at inclusion in the Epidemiological Investigation of Rheumatoid Arthritis (EIRA), with nulliparous women as reference. *ACPA* Anti-citrullinated protein antibody, *CI* confidence interval, *DAS28* Disease activity score 28, *HAQ* Health assessment questionnaire

No association between parity and severity of ACPA-positive disease was observed (Table 2).

### Parity and severity of ACPA-negative RA at different time points: analysis of covariance

Since parity only had an impact on the outcome measures in the ACPA-negative subset, we limited further analyses to that group and compared mean differences at each follow-up visit (Table 3).

**Table 3:** Analysis of covariance with mean differences of outcome measures (DAS28, HAQ) between nulliparous and parous women with incident RA included in the EIRA study, at baseline and follow-up visits during the first 24 months

Outcome measure above median	ACPA-negative RA	
	18–44 years	45–70 years
	Beta (95 % CI), *p*-value	Beta (95 % CI), *p*-value
DAS28		
Baseline	0.02 (−0.58 to 0.62), *p* = 0.95	−0.78 (−1.19 to −0.37), *p* < 0.001
3 months	1.60 (0.81 to 2.40), *p* < 0.001	−0.45 (−0.96 to 0.06), *p* = 0.96
6 months	1.30 (0.48 to 2.12), *p* = 0.002	−0.27 (−0.82 to 0.28), *p* = 0.34
12 months	1.22 (0.59 to 1.85), *P* < 0.001	−0.31 (−0.73 to 0.12), *p* = 0.16
24 months	0.86 (0.18 to 1.55), *p* = 0.014	−0.29 (−0.76 to 0.19), *p* = 0.24
HAQ		
Baseline	0.06 (−0.23 to 0.34), *p* = 0.68	−0.35 (−0.56 to −0.14), *p* = 0.001
3 months	0.40 (0.05 to 0.74), *p* = 0.026	−0.12 (−0.32 to 0.08), *p* = 0.24
6 months	0.44 (0.13 to 0.75), *p* = 0.006	−0.09 (−0.30 to 0.12), *p* = 0.38
12 months	0.43 (0.16 to 0.69), *p* = 0.002	−0.02 (−0.20 to 0.16), *p* = 0.80
24 months	0.48 (0.14 to 0.82), *p* = 0.006	−0.17 (−0.38 to 0.05), *p* = 0.13

Analysis of covariance adjusted for age at inclusion in the Epidemiological Investigation of Rheumatoid Arthritis (EIRA), with nulliparous women as reference. *ACPA* Anti-citrullinated protein antibody, *CI* confidence interval, *DAS28* Disease activity score 28, *HAQ* health assessment questionnaire, *RA* Rheumatoid arthritis

Parous women in the younger ACPA-negative group had significantly higher levels of HAQ and DAS28 in all repeated measurements except at baseline, where there were no differences. Among those aged >45 years at inclusion, parous women had lower levels of DAS28 and HAQ compared to nulliparous women at all time points, although the difference only reached statistical significance at baseline.

### High verus low disease severity: logistic regression analysis

Parous women who developed ACPA-negative disease at reproductive age had higher odds of having a DAS28 and HAQ value above the median compared to nulliparous women at all follow-up visits, with significance reached at 3, 12 and 24 months for DAS28 and at 12 months for HAQ. An indication of an opposite effect was seen in the ACPA-negative older group, especially at baseline (Table 4).

**Table 4:** Logistic regression for parous women compared to nulliparous women

Outcome measure above median	ACPA-negative RA	
	Aged 18 − 44	Aged 45 − 70
	OR (95 % CI), *p*-value	OR (95 % CI), *p*-value
DAS28		
Baseline	1.87 (0.62 to 5.69), *p* = 0.27	0.37 (0.18 to 0.80), *p* = 0.011
3 months	4.96 (1.37 to 18.1), *p* = 0.015	0.51 (0.24 to 1.08), *p* = 0.08
6 months	2.66 (0.78 to 9.15), *p* = 0.12	0.37 (0.16 to 0.85), *p* = 0.018
12 months	4.49 (1.37 to 14.7), *p* = 0.013	0.89 (0.44 to 1.77), *p* = 0.97
24 months	3.56 (1.07 to 11.9), *p* = 0.039	0.67 (0.30 to 1.50), *p* = 0.33
HAQ		
Baseline	0.58 (0.20 to 1.69), *p* = 0.32	0.53 (0.26 to 1.07), *p* = 0.08
3 months	3.04 (0.90 to 10.3), *p* = 0.07	1.29 (0.62 to 2.67), *p* = 0.50
6 months	2.42 (0.71 to 8.23), *p* = 0.16	1.01 (0.47 to 2.14), *p* = 0.99
12 months	7.92 (2.02 to 31.0), *p* = 0.003	1.15 (0.57 to 2.33), *p* = 0.69
24 months	2.14 (0.65 to 7.06), *p* = 0.21	0.95 (0.42 to 2.17), *p* = 0.90

Logistic regression adjusted for age at inclusion in the Epidemiological Investigation of Rheumatoid Arthritis (EIRA), with the odds ratio (OR) of having an outcome measure (DAS28 and HAQ) higher than median for a parous woman. *ACPA* Anti-citrullinated protein antibody, *CI* confidence interval, *DAS28* Disease activity score 28, *HAQ* Health assessment questionnaire

## Discussion

In this study, we demonstrated that parity might have an impact on disease activity and disability in ACPA-negative disease. In those who developed RA at reproductive age (18–44 years), a more severe clinical outcome, measured with DAS28 and HAQ, was observed among parous as compared to nulliparous women. In the older age group (45–70 years at inclusion) we observed a milder disease in parous women, though only statistically significant at baseline. There was no association between parity and the severity of ACPA-positive disease, neither among younger nor among older women.

In a recent study we showed that parous women of reproductive age (18–44) had a higher risk of ACPA-negative disease, and we now show that their disease course also seems to be more severe [10]. Regarding the effect of parity on RA severity over time, there are two previous studies with somewhat diverging results [13, 14]. However, this is the first study investigating the association between parity and the severity of RA with stratification for ACPA status.

Strengths of this study include the frequent follow-up program and the large sample size, which allows us to perform stratified analyses of different RA subgroups, as well as different age groups. The inclusion of only incident cases also ascertains that parity was not affected by the disease.

One limitation was the lack of information about parity after diagnosis. If ACPA-negative women had more pregnancies after disease onset it might have affected our results on disease severity over time. However, the number of women who might have become pregnant within the 2-year follow-up time is probably limited (178 women were in the age group 18–35 years). Another limitation was the lack of disease severity parameters at baseline in a subset of patients. In a failure analysis, no major differences in parity (78 versus 82 % in the presented population) or university degree (26 versus 26 %) were found for those included in EIRA only compared to those included in both EIRA and SRQ.

Hypothetically, our findings may be explained by immunomodulatory changes related to pregnancy, for example a decrease of anti-inflammatory cytokines [20] and regulatory CD4+ T cells [21] postpartum, which could drive the progression of ACPA-negative RA in younger women, especially close to delivery. In a study of a population of North American natives highly predisposed to RA, the highest incidence of RA was observed in the first postpartum year [22]. This suggests that reversal of hormonal and immunologic changes during pregnancy that favor immune tolerance (and prevent maternal rejection of the fetus) may affect RA development and increase early disease severity.

The impact of parity on disease activity and disability in ACPA-negative disease among women aged 18–44 years might be confined to the postpartum period. However, since there were too few observations to study RA progression directly after delivery, we included all women aged 18–44 years in the analysis. Another hypothesis is that ACPA-negative younger women who had been pregnant could have poorer response to treatment or lesser compliance to prescribed medications than ACPA-negative women who had not yet been pregnant, certainly if they are planning new pregnancies. However, we cannot exclude that women who had not been pregnant before diagnosis also are planning pregnancy in the near future.

Other possible explanations include an impact of sleep disturbance and other factors that could influence pain in the postpartum period. This should be further studied.

In older women, the influence of parity may reflect the influence of related lifestyle factors, or a long-term impact of hormone-related factors, which have been shown to be more important for seronegative RA [23].

## Conclusion

Parity may be a predictor of higher DAS28 and HAQ compared to nulliparity in ACPA-negative RA women of reproductive age at diagnosis, but seemed to be a predictor of less severe disease amongst older women, at least at baseline. Parity did not seem to be a predictor of severity in ACPA-positive disease. These findings indicate that immunomodulatory changes during and after pregnancy may have specific impact on the ACPA-negative RA phenotype. Further studies of the underlying mechanisms may be the basis for a particular management of ACPA-negative younger women with RA in the future.

## Abbreviations

ACPA: Anti-citrullinated protein antibodies
CI: confidence interval
DAS28: Disease activity score 28
EIRA: Epidemiological Investigation of Rheumatoid Arthritis
HAQ: Health assessment questionnaire
RA: Rheumatoid arthritis
SRQ: Swedish Rheumatology Quality Register
